# EGL-4 promotes turning behavior of *C. elegans* males during mating

**DOI:** 10.17912/micropub.biology.000433

**Published:** 2021-08-18

**Authors:** Shapour Rahmani, Simon Tuck

**Affiliations:** 1 Umeå Center for Molecular Medicine, Umeå University, Sweden

## Abstract

During mating, *C. elegans* males whose tails have reached the head or tail of their intended mates are able to switch to scanning the other side by performing a turn during which the male’s tail curls ventrally all the while keeping in contact with the hermaphrodite. The ability to execute turns efficiently is dependent upon serotonergic neurons in the posterior ventral nerve cord that stimulate diagonal muscles by activating a serotonin receptor, SER-1. Here we show that turning behavior is abnormal in males lacking a cGMP-dependent protein kinase, EGL-4. *egl-4* mutant males are also resistant to ventral tail curling induced by exogenous serotonin.

**Figure 1:**
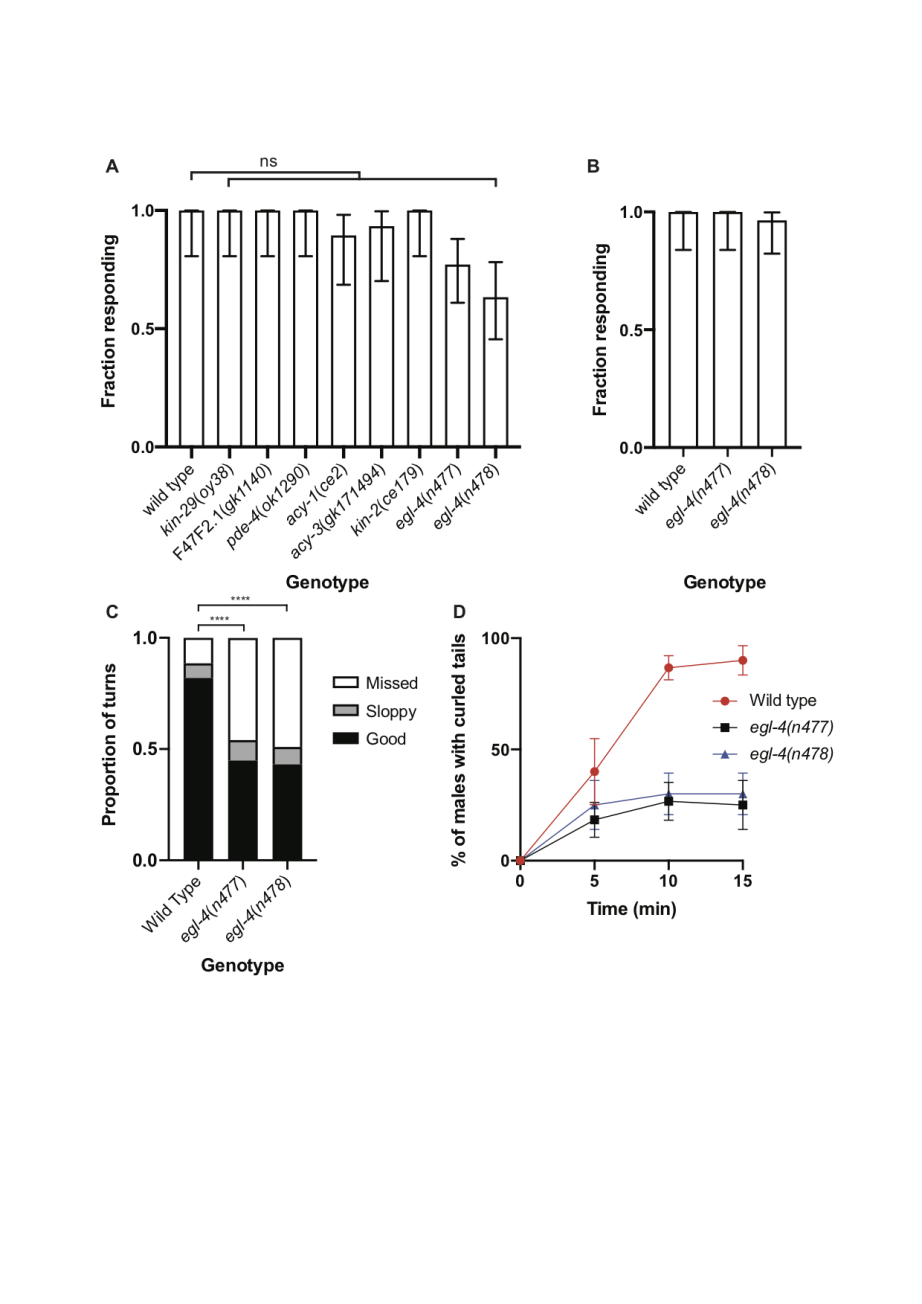
A. Graph showing the fraction of males of the genotypes indicated that responded to wild-type hermaphrodites within 10 minutes. Error bars indicate 95% confidence intervals. At least 16 males of each genotype were assayed. For these assays, males were placed on plates containing 10 wild-type hermaphrodites. B. Graph showing the fraction of *egl-4* mutant males that responded when males were placed on plates containing 30 hermaphrodites. Error bars indicate 95% confidence intervals. C. Graph showing the proportion of turns initiated by males of the indicated genotypes that were successful. The criteria used to judge turns as good, sloppy or missed were those described previously (Loer and Kenyon, 1993): A turn was judged to be “good” if the male’s tail was in continuous contact with the hermaphrodite throughout the turn. A “sloppy” turn is defined as a turn in which the male succeeded in making the turn but the tail lost contact with the hermaphrodite during the turn. A turn is said to have been missed if the male’s tail sailed off the end of the hermaphrodite and contact was not reestablished. **** indicates P<0.0001 (Fisher’ exact test). For turning assays, 30 hermaphrodites were placed on a thin bacterial lawn generated with 13 μl of a culture of OP50 bacteria. Under these conditions, 20/20 *egl-4*(*n477*) and 27/28 *egl-4*(*n478*) males responded to an hermaphrodite within 10 minutes and most responded within five minutes. D. Graph showing the percentage of males with ventrally curled tails at different time points after the addition of serotonin. Males were placed in M9 medium in the wells of microtiter plates and serotonin hydrochloride was added to give a final concentration of 20 mM. 10 males were placed in each well. A total of 60 animals was analyzed for each genotype. Error bars indicate 95% confidence intervals.

## Description

*C. elegans* males that have come into close proximity of hermaphrodites initiate copulatory behavior comprising at least five different steps termed response, turning, location of vulva, spicule insertion and sperm transfer (Loer and Kenyon 1993, Liu and Sternberg 1995, Chute and Srinivasan 2014). Mutations specifically affecting different steps have been isolated and characterized (Barr and Sternberg 1999, Hajdu-Cronin *et al.* 2017, Liu *et al.* 2017). However, our understanding of the molecular mechanisms acting in the neurons controlling copulation is far from complete. During the response step, males that have sensed the presence of a hermaphrodite move backwards in such a way that the male’s tail fan glides along the surface of the hermaphrodite until the tail reaches the vulva (or head or tail) (Loer and Kenyon 1993, Liu and Sternberg 1995, Sherlekar and Lints 2014). Response behavior is regulated by ciliated neurons in the tail whose dendrites lie in sensory rays within the fan (Liu and Sternberg 1995). If a male reaches the end of the hermaphrodite without having found the vulva, it executes a turn during which the tail bends tightly ventrally so that contact is established between the ventral surface of the fan and the other side of the intended mate (Loer and Kenyon 1993, Liu and Sternberg 1995). The ability to execute turns efficiently is dependent upon serotonergic neurons in the posterior ventral nerve cord (the CP neurons) and on their ability to produce serotonin (Loer and Kenyon 1993, Carnell *et al.* 2005). Serotonin stimulates the diagonal muscles in the tail to induce curling ventrally by stimulating a serotonin receptor, SER-1 (Loer and Kenyon 1993, Carnell *et al.* 2005). However, how serotonin affects diagonal muscles and ventral turning is not fully understood.

Characterization of the neural networks controlling copulation have indicated that many of the neurons involved have overlapping functions (Liu and Sternberg 1995, Sherlekar and Lints 2014). Furthermore, genetic analysis of copulation has suggested that some of the steps might be regulated by pathways that are genetically redundant. Thus, mutations affecting different steps in copulation might be missed in forward genetic screens. The activities of some sensory neurons in *C. elegans* and other species are modulated by the cyclic nucleotides cAMP or cGMP (Koelle 2018). An important effector of cAMP is cAMP-dependent protein kinase (protein kinase A), whose regulatory subunit is encoded in *C. elegans* by *kin-2*. However, *kin-2* mutant males as well those mutant for *kin-29*, F47F2.1 or *pde-4*, which all affect cAMP-dependent signaling, were not obviously defective in their response to hermaphrodites (Fig. 1A). An important effector of cGMP in *C. elegans* is EGL-4, a cGMP-dependent protein kinase (Daniels *et al.* 2000, Hirose *et al.* 2003). *egl-4* loss-of-function mutant males tended to respond slightly less efficiently than wild-type males in standard response assays (Fig. 1A) but in the presence of a large excess of hermaphrodites almost all *egl-4* mutant males were able to respond (Fig. 1B). However, while conducting these assays we noticed that the *egl-4* loss-of-function mutant males were defective in their turning behavior and frequently became detached from the hermaphrodite while attempting turns. Quantification of this defect revealed that, compared to those performed by wild-type males, many more of the attempted turns made by *egl-4* mutant males were aberrant (Fig. 1C). The turning defect of *egl-4* mutant males is similar to that displayed by *cat-1* mutant males, which have a reduced ability to synthesize serotonin (Loer and Kenyon 1993). Aberrant turning behavior likely contributes to the reduced ability of *egl-4* mutant males to sire progeny in crosses (Daniels *et al.* 2000).

Application of exogenous serotonin to wild-type males induces tight ventral curling of the tail (Loer and Kenyon 1993). In hermaphrodites, serotonin signaling induces egg-laying and a behavioral state termed dwelling characterized by (among other things) reduced locomotion (Trent *et al.* 1983, Flavell *et al.* 2013). Both egg-laying and dwelling behavior are also regulated by *egl-4* (Fujiwara *et al.* 2002, Raizen *et al.* 2006). Furthermore, extended exposure of wild-type hermaphrodites to serotonin induces paralysis but *egl-4* loss-of-function mutant hermaphrodites are partially resistant to paralysis (Olson and Koelle 2019). Consistent with these results, induction of tail curling by exogenous serotonin was less efficient in *egl-4* mutant males than in wild type (Fig. 1D). It has previously been shown that whereas SER-1 promotes tail curling, a serotonin-gated chloride channel, MOD-1, has the opposite effect (Carnell *et al.* 2005). Similarly, *mod-5* mutant hermaphrodites are hypersensitive to serotonin-induced paralysis (Olson and Koelle 2019). Thus, models in which EGL-4 either promotes SER-1 activity in diagonal muscles or inhibits MOD-1 activity are consistent with our observations. A further possibility is that EGL-4 acts in a pathway activated by SER-1.

## Methods

**Male response and turning assays**

Males of all genotypes were obtained by RNAi of *him-8* and *klp-16* (Timmons *et al.* 2014). Turning behaviour, and the response of males to hermaphrodites were assessed by procedures described previously (Loer and Kenyon 1993, Liu and Sternberg 1995). In both cases, wild-type hermaphrodites were used in the assays. For the turning assays, more than 60 turns/attempted turns were observed for each genotype. More than 20 different males were observed for each genotype. The analysis of tail curling in response to exogenous serotonin was carried out as previously described (Loer and Kenyon 1993). Males were placed in the wells of a microtiter plate and serotonin hydrochloride (obtained from Sigma-Aldrich) was added to give a final concentration of 20 mM.

## Reagents

Strain list.

**Table:** 

Strain	Genotype	Available from
N2	*Caenorhabditis elegans*	CGC
MT1072	*egl-4*(*n477*)	CGC
MT1073	*egl-4*(*n478*)	CGC
KG518	*acy-1*(*ce2*)	CGC
VC2531	F47F2.1(*gk1140*)	CGC
KG532	*kin-2*(*ce179*)	CGC
PY1479	*kin-29*(*oy38*)	CGC
RB1231	*pde-4*(*ok1290*)	CGC
VC20501^a^	*acy-3*(*gk171494*)	CGC

^a^This strain is from the million-mutation project and contains mutations in other genes.
